# Application of Broad-Spectrum, Sequence-Based Pathogen Identification in an Urban Population

**DOI:** 10.1371/journal.pone.0000419

**Published:** 2007-05-09

**Authors:** Baochuan Lin, Anthony P. Malanoski, Zheng Wang, Kate M. Blaney, Adam G. Ligler, Robb K. Rowley, Eric H. Hanson, Erik von Rosenvinge, Frances S. Ligler, Anne W. Kusterbeck, David Metzgar, Christopher P. Barrozo, Kevin L. Russell, Clark Tibbetts, Joel M. Schnur, David A. Stenger

**Affiliations:** 1 Center for Bio/Molecular Science and Engineering, Code 6900, Naval Research Laboratory, Washington, D. C., United States of America; 2 NOVA Research Incorporated, Alexandria, Virginia, United States of America; 3 Headquarters United States Air Force/Surgeon General, Falls Church, Virginia, United States of America; 4 Malcolm Grow USAF Medical Center, Andrews Air Force Base, Maryland, United States of America; 5 Department of Defense Center for Deployment Health Research, Naval Health Research Center, San Diego, California, United States of America; University of Liverpool, United Kingdom

## Abstract

A broad spectrum detection platform that provides sequence level resolution of target regions would have a significant impact in public health, case management, and means of expanding our understanding of the etiology of diseases. A previously developed respiratory pathogen microarray (RPM v.1) demonstrated the capability of this platform for this purpose. This newly developed RPM v.1 was used to analyze 424 well-characterized nasal wash specimens from patients presenting with febrile respiratory illness in the Washington, D. C. metropolitan region. For each specimen, the RPM v.1 results were compared against composite reference assay (viral and bacterial culture and, where appropriate, RT-PCR/PCR) results. Across this panel, the RPM assay showed ≥98% overall agreement for all the organisms detected compared with reference methods. Additionally, the RPM v.1 results provide sequence information which allowed phylogenetic classification of circulating influenza A viruses in ∼250 clinical specimens, and allowed monitoring the genetic variation as well as antigenic variability prediction. Multiple pathogens (2–4) were detected in 58 specimens (13.7%) with notably increased abundances of respiratory colonizers (esp. *S. pneumoniae*) during viral infection. This first-ever comparison of a broad-spectrum viral and bacterial identification technology of this type against a large battery of conventional “gold standard” assays confirms the utility of the approach for both medical surveillance and investigations of complex etiologies of illness caused by respiratory co-infections.

## Introduction

Emerging infections with nonspecific symptoms have enormous impact on public health and global economics (e.g. severe acute respiratory syndrome outbreaks, and avian (H5N1) influenza). Such outbreaks emphasize the need for efficient simultaneous detection of multiple organisms that will facilitate timely and judicious implementation of countermeasures for outbreak containment, treatment interventions, and case management [1]. Ideally, a system capable of detecting all potentially relevant organisms is needed. There are many technologies being developed with a view toward addressing this issue, for example MasscodeTM multiplex RT-PCR system [2], electrospray ionization mass spectrometry analysis of PCR amplicons [3], Luminex® xMAP™ [4], and other microarray based approaches [5], [6], [7], [8], [9], [10], [11]. While promising, these technologies have only been tested for detection of a few pathogens or particular classes of pathogens simultaneously. In addition, these technologies provide a very limited range of genetic resolution and require additional testing for determination of detailed mutation or strain-variations in detected pathogens. High density resequencing arrays provide resolution of individual nucleotides in long target sequences (hundreds to thousands of nucleotides) [12], [13]. We have demonstrated discrimination of mutations in targeted pathogens as well as sensitivities and specificities that are similar to or improved over those of other technologies based on a subset of the organisms the microarray is designed to detect. The respiratory pathogen microarray version 1 (RPM v.1), while not providing comprehensive coverage of all potential causes of respiratory illness, targets a much broader range of organisms (including bacteria and DNA and RNA viruses) in a single test than other potential methods [14], [15], [16], [17].

Our previous work on the RPM v.1 demonstrated detection of pathogens individually at 10^1^–10^3^ genome copies using spiked samples as well as select organisms in clinical samples. We have also demonstrated the use of culture isolates of clinical samples in which a more detailed analysis of strain distribution in influenza viruses was possible [17]. Unfortunately, when directly using clinical samples, only the successful attribution of flu samples to the correct flu season was accomplished [15]. The accuracy of the base calls from the array has been compared with only *de novo* sequencing using culture isolates of clinical samples. Our studies demonstrate that one of the challenges in developing multiplex detection systems for a broad range of very different organisms is the validation of the test results. Few samples are available that have been subjected to testing for a large number of diverse pathogens. Similarly, the archived clinical specimens used in our previous studies were originally only tested for a few select targets. When multiple organisms were detected by the RPM v.1, it was not always possible to perform confirmatory testing due to a lack of available materials. This is a limitation of using archived samples for validating newly developed multiplex assays as it is very time-consuming and costly thus making multiple confirmatory assays in clinical specimens very difficult to achieve.

In this study, we validate the potential of the respiratory pathogen microarray (RPM v.1) for providing broad-spectrum pathogen detection. A set of 424 clinical samples collected in the Washington, DC metropolitan region from December 2004 to February 2005 were subjected to a full panel of conventional microbial analysis (culture and/or RT-PCR/PCR) as well as analysis by the RPM v.1 microarray. This set of samples demonstrated good agreement across the panel of tests making authentication of multiple detection events by the RPM v.1 microarray possible. Many specimens from this set represented co-infections providing a range of multi-target samples for analysis. Here we use clinical samples rather than culture isolates and demonstrate detailed analysis of strain distributions of influenza viruses within a flu season as well as providing a phylogenetic measure of circulating influenza strains. The accuracy of the base calls was confirmed by comparison to *de novo* sequencing using representative clinical samples. This comparison of a broad-spectrum viral and bacterial identification technology with a large battery of conventional “gold standard” assays represents more rigorous testing of our approach for both medical surveillance and investigation of complex etiologies than has been carried out previously. This study provides a previously unavailable fundamental assessment of a multiple pathogen detection assay and paves the way to improvements for a microarray-based platform which would provide more comprehensive multiple pathogen detection in a single test. The implications and limitations of the assay are also discussed.

## Methods

### Specimen collection and processing

Specimens were collected at six military treatment facilities in the Washington, DC metropolitan region between December 2004 and February 2005. The demographics of the Washington, DC metropolitan region have previously been shown to have a broad age, gender, and geographic distribution [18]. Patients were recruited from both emergency department and clinic settings, after the nature and possible consequences of the study were explained and informed consents were obtained from participants. Patient samples were stored in CVM media (Hardy Diagnostics, Santa Maria, CA) for viral culture processing, or stored in tryptic soy broth with glycerol for bacterial analysis, and were shipped frozen to destination laboratories for analysis. These samples were collected, and this research has been conducted in compliance with all applicable federal and international regulations governing the protection of human subjects in research, under Naval Medical Center protocols #B05LHOOOOO-018.

### Microarray hybridization and analysis

The RPM v.1 design and specimen process protocols for microarray analysis were described in detail in previous publications [14], [15], [16], [17]. Microarray hybridization and processing were carried out according to the manufacturer's recommended protocol (Affymetrix Inc., Santa Clara, CA) with the following modifications: Purified PCR products were fragmented at 37°C for 5 minutes, and then labeled with Biotin-N6-ddATP at 37°C for 30 minutes. Hybridization was carried out in the hybridization oven at 45°C and 60 rev/min for 4 hours. The image scanning and processing to produce FASTA output file were performed as previously described [16]. Final pathogen identification for the RPM v.1 assay was performed using Computer-Implemented Biological Sequence Identifier (CIBSI) Version 2.0 software [19], an automatic pathogen identification algorithm (based on nucleic acid sequence) that was developed and tested in detail in previous studies [16], [20].

### Reference assays

Independent assays were performed using bacterial and viral culture except for difficult-to-culture pathogens, for which PCR methods were used. Additional assays, primarily conventional and/or real-time PCR and RT/PCR, were performed on culture negative samples to test for pathogens occurring at titers too low for culture and/or loss of viability from transportation. Overall, a specimen was defined as “positive” for a pathogen if the composite assay results (culture and/or duplicate PCR assays) were positive.

Viral analyses were performed by FOCUS Diagnostics, Inc., a CAP-certified reference laboratory (Cypress, CA) or by Air Force Institute for Operational Health (Brooks City Base, TX). Viral processing included: inoculation of sample, monitoring for cytopathic effect (7–15 days of monitoring), sub-passage as necessary and confirmatory testing as needed (i.e., direct monoclonal immunofluorescence assay, indirect immunofluorescence assay or PCR).

Bacterial analyses were performed at the Naval Health Research Center (NHRC, San Diego, CA) Respiratory Disease Laboratory. Samples were then analyzed for the presence of bacterial pathogens utilizing classic culture techniques with selective agar plates. For all culture methods, if growth resembled colonies appropriate to a targeted pathogen, confirmatory tests (e.g., susceptibility disks, biochemical testing) were conducted to verify the identity of the suspected colony.

Initial PCR analyses of difficult-to-culture pathogens (*Bordetella pertussis, Chlamydia pneumoniae/psittaci*, coronavirus OC43 and 229E, and *Mycoplasma pneumoniae)* were performed at the NHRC Respiratory Disease Laboratory or FOCUS Diagnostics, Inc. (Cypress, CA). Further species-specific RT/PCR amplification assays were performed on culture-negative but RPM v.1-positive samples with positive (culture-positive, RPM v.1-positive) and negative (culture-negative, RPM v.1-negative) controls. These assays were performed at NRL using previously published primers (Table S1). In general, 25 µl PCR reactions were run on MyiQ™ real-time PCR detection systems (Bio-Rad, Hercules, CA, USA). Conventional PCR assays were analyzed by electrophoresis thorugh1.5% TAE agarose gels. Sequencing reactions were performed by SequeTech (Mountain View, CA).

Sequence alignments for influenza-positive samples were performed on *HA3 hemagglutinin* sequences using MagAlign function of Lasergene version 6 (DNASTAR, Inc. Madison, WI). Rooted phylogenetic trees were generated by using neighbor-joining method in PAUP* 4.0 (Sinauer Associates, Inc., Publishers, Sunderland, MA) and rooted to A/Panama/2007/99. Reliability estimates were assessed using 1,000 bootstrap replicates.

## Results

A total of 424 samples were tested using RPM v.1 assays and parallel reference assays (culture and/or PCR tests). The sensitivity and specificity of the RPM v.1 assay were determined using various prototype strains and clinical samples in a previous study [15]. Table 1 shows the collective number of positive results obtained using culture assays only, all reference assays, and RPM v.1. Of the 424 samples, 269 (63.4%) were identified as positive for influenza A by RPM v.1, compared to 176 (41.5%) by culture (30 to 42-day). Similarly, 46 (10.8%) were positive for influenza B using RPM v.1, while culture identified 28 (6.6%) as influenza B-positive. RPM v.1 identified 24 (5.6%) as positive for coronavirus (229E or OC43), while PCR identified 28 (6.6%) samples as coronavirus (229E and OC43) positive. Additional pathogens were identified in low incidence–shown here as [RPM v.1 vs. culture], including adenovirus-[9 (2.1%) vs. 2 (0.5%)], *Streptococcus pneumoniae*-[38 (8.9%) vs. 8 (1.9%)], and *S. pyogenes*-[13 (3.1%) vs. 9 (2.1%)]. RPM v.1 also identified type I and III parainfluenza (PIV) virus, PIV1 (1, 0.2%), PIV3 (2, 0.5%), rhinovirus type 89 (2, 0.5%), *M. pneumoniae* (3, 0.7%), and *Neisseria meningitides* (14, 3.3%) which were not identified by culture. The RPM v.1 did not contain resequencing tiles for PIV2 or *Haemophilus influenzae,* both of which were detected by culture, PIV2 (2, 0.5%) and *H. influenzae* (7; 1.7%).

**Table 1:** Pathogens identified for 424 matched specimens-overall microarray vs. reference methods.

Organism	Culture (+)	Ref^©^ (+)	RPM v.1 (+)	Ref^©^ (+), RPM v.1 (−)	Ref^©^ (−), RPM v.1 (+)
Adenovirus	2	8	9	0	1
Coronavirus	28*	29	24	6	2
Influenza A	176	263	269	1	7
Influenza B	28	41	46	1	6
PIV 1	0	0	1	0	1
PIV3	0	0	2	0	2
Rhinovirus	0	1	2	1	1
*M. pneumoniae*	0	2	3	0	1
*S. pneumoniae*	8	40	38	3	1
*S. pyogenes*	9	13	13	0	0
Negative	176	52	59	0	7

Note: *Coronaviruses were identified through CAP-certified PCR method, Ref^©^: reference assays-culture and/or RT-PCR/PCR positive.

In this study, influenza virus was the most commonly identified respiratory pathogen by all methods. With respect to all reference assays, the RPM v.1 method showed a detection sensitivity of 99% and a specificity of 96%, and an overall agreement of 98% (Table 2) for influenza A virus. For influenza B, the RPM v.1 detection sensitivity and specificity were 98% with an overall agreement of 97% (Table 2). All but three influenza culture-positive specimens were also positive on microarrays. However, two specimens identified as influenza A-positive by culture were clearly detected as influenza B on microarrays. Real-time RT-PCR later concurred with the microarray results and not culture that these 2 specimens were indeed influenza B-positive. The RPM v.1 also demonstrated excellent detection sensitivity, specificity, and overall agreement with respect to the reference assay results for other pathogens detected (Table S2–S3).

**Table 2:** Evaluation of the detection efficiency for influenza A and B viruses in clinical samples

	*Influenza A*		*Influenza B*	
	Ref^©^+	Ref^©^−	Ref ^©^+	Ref^©^−
RPM v.1+	262	7	40	6
RPM v.1−	2	153	1	377
Sensitivity	99%		98%	
Specificity	96%		98%	
Overall agreement	98%		98%	

Ref^©^: reference assays-culture and/or PCR

The capability of the RPM v.1 system for the identification of complex mixtures of pathogens [15], [16], [17] were further demonstrated through assessment of the incidence of co-infections in the 424 samples. Of these samples, 58 (13.7%) showed viral or viral/bacterial co-infections as determined by RPM and/or culture (Table 4). These co-infections were further verified using published type-specific PCR assays and in-house specific PCR primers (Table S1). It is well known that *S. pneumoniae* and *N. meningitidis* colonize the mouth and upper respiratory system, so it is not surprising that these were common co-infections found in clinical samples. However, consistent with our previous study [15], quantitative real-time PCR data showed that most of the *S. pneumoniae* present in influenza-positive samples harbored a high titer (≥10^4^ genome copies/µl) as compared to influenza-negative samples (data not shown). The high titer bacteria present in these clinical samples was possibly virally induced bacterial superinfection, as first suggested by the findings of Madhi et al. [21] and Peltola and McCullers [22].

A critical aspect for influenza epidemiology is to track genetic changes within influenza strains, since antigenic drift is the mechanism by which influenza viruses escape from immunological pressure induced by previous natural exposures and vaccinations. Analysis of the key amino acids (deduced from nucleotide sequence) in the HA3 sequences of all influenza A H3N2 positive isolates revealed two major circulating strains: A/New York/258/2005 (Group I) and A/Aichi/133/2005 (Group II) (Table 3). Group I belongs to the A/California/7/2004 lineage and carries signature amino acids substitutions in antigenic site D: valine to isoleucine at position 226 (V226I) and serine to proline at position 227 (S227P). Group II showed an A/Wellington/1/04 lineage signature amino acid substitution at position 227 (S227P), and serine to asparagine at position 216 (S216N), the key amino acid at antigenic site B. Surprisingly, the only outlier sample was identified as A/Wyoming/3/03 (with IS)-like isolate. Amino acids position at 216 correspond to 188, 226 corresponds to 198, and 227 corresponds to 199 on the tiled prototype sequence (Table 3).

**Table 3:** Nucleotides difference in *hemagglutinin* (HA3) genes identified by RPM v.1 from 250 influenza A/H3N2 isolates.

Position aa/nt*	Amino acid and nucleotide substitution^&^						
	A/Fujian/411/02^#^	A/Wyoming/3/03		A/Wellington/1/04		A/California/7/04	
8 (36)/25	Val/GTT	Val/GTT	1	Val/GTC	71	Val/GTT	166
28 (56)/83	His/CAT	His/CAT	1	His/CAT	56	His/CAT	166
				Tyr/TAT	15		
39 (67)/116	Ile/ATA	Val/GTA	1	Ile/ATA	71	Ile/ATA	167
81(109)/244	Arg/AGG	Arg/AGG	1	Arg/AGA	64	Arg/AGG	161
				Arg/AGG	3		
100 (128)/299	Ala/GCT	Thr/ACT	0	Thr/ACT	33	Thr/ACT	90
117 (145)/352	Lys/AAA	Asn/AAC	1	Asn/AAC	62	Asn/AAC	144
131 (159)/393	Tyr/TAC	Phe/TTC	1	Phe/TTC	65	Phe/TTC	159
149 (177)/446	Leu/TTG	Leu/TTG	1	Leu/CTG	39	Leu/TTG	144
161 (189)/483	Ser/AGT	Asn/AAT	1	Asn/AAT	67	Asn/AAT	152
188 (216)/564	Asn/AAT	Asn/AAT	1	Ser/AGT	67	Asn/AAT	144
						Ser/AGT	1
195 (223)/584	Val/GTA	Val/GTA	1	Ile/ATA	59	Val/GTA	160
198 (226)/593	Val/GTC	Ile/ATC	1	Val/GTC	61	Ile/ATC	43
199 (227)/596	Ser/TCC	Ser/TCC	1	Pro/CCC	66	Pro/CCC	5

Note: * Amino acid (aa) and nucleotide (nt) positions correspond to the “prototype sequence” for *hemagglutinin* of influenza A/H3N2 (HA3) on RPM v.1; the number in the parenthesis correspond to the position of full length HA3 sequence, ^&^ amino acid and its corresponding codon at each position were separated by “/”. SNPs relative to the prototype sequence are underlined. The numbers in column 4, 6, and 8 indicate the number of samples containing the amino acid substitution; ^#^HA3 prototype sequence was derived from A/Fujian/411/02 strain. A/New York/258/2005 (California-lineage) and A/Aichi/133/2005 (Wellington-lineage) represent two major groups identified from all isolates. The one outlier was identified as A/Wyoming/3/03 (with IS)-like isolate.

**Figure 1:**
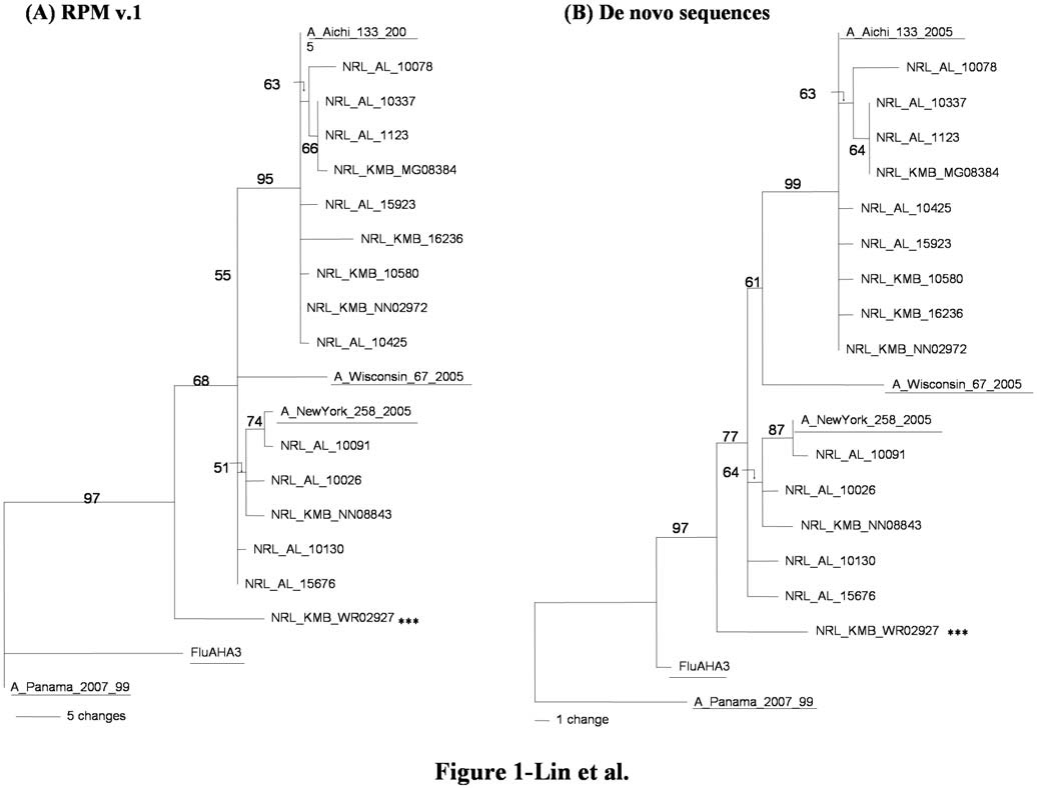
Rooted phylogenetic analysis of the *hemagglutinin* (HA3) genes of 15 representative influenza A/H3N2 isolates and reference strains. (A) phylogenetic tree generated using sequences obtained from RPM v.1, (B) phylogenetic tree generated using de novo sequences from the same set of the isolates. Reference sequences were obtained from GenBank and indicated in underlined, bold font. Numbers above branches indicate bootstrap values from 1000 replicates. Note: ***-the A/Wyoming/3/03 (with IS)-like isolate. Bootstrap values above 50% were shown at branches. Scale near the bottom of each panel relates the length of a branch to the number of nucleic acid substitutions.

Using 15 representative samples, phylogenetic analysis comparison between the sequences generated from RPM v.1 (68 to 96% resolved bases) (Fig. 1A) and the sequences generated via conventional sequencing (100% resolved bases) (Fig. 1B), showed similar results, indicating that ambiguous base calls from the microarray did not affect phylogeny determination for influenza A viruses (Table 3, Fig. 1). The representative samples were the A/Wyoming/3/03 (with IS)-like isolate and 14 other samples randomly selected from the two groups. The resulting phylogenetic trees clearly confirmed that the influenza A/H3N2 positive samples consisted of two major groups: A/New York/258/2005 (A/California/7/2004 lineage) and A/Aichi/133/2005 (A/Wellington/1/04 lineage) with one A/Wyoming/3/03 (with IS)-like isolate as an outlier. Further confirmation using all HA3 sequences produced the same groups that were identified by tracking key amino acid substitutions (data not shown). Both groups apparently originated from the common ancestor A/Fujian/411/2002 lineage. The Wellington-like strains had longer distances than New York-like strains suggesting that Wellington-like strains had more genetic variations and they might be evolved from California lineage. For influenza B, the results showed that all the isolates were close to the B/Texas/3/2002 strain, which belongs to B/Yamagata/16/88 lineage and was the circulating strain from the 2004–2005 influenza season.

## Discussion

Despite the effort to establish completely blind independent testing for every pathogen (viral and bacterial culture and, where appropriate, RT-PCR/PCR), differences in detection sensitivities of the methods and transportation issues necessitates using further confirmatory testing. This is because a measure of uncertainty is introduced in the quality of the reference assays as the services of a local reference laboratory could not be arranged (samples were transported to San Diego, CA). Furthermore, although large sample volumes are set aside for reference assays, a reduction in detection sensitivity is still inevitable because these samples are split and frozen before testing for each viral and bacterial agent in separate cultures. To reduce this uncertainty, archived samples stored at the time of the original testing are retested using real-time RT-PCR/PCR in house as an additional reference method on RPM-positive but culture-negative samples, using positive (positive for both RPM and culture) and negative controls (negative for both RPM and culture). Combining the results of all confirmatory tests demonstrates an excellent concordance with RPM v.1 (Table 2 and Table S2–S3) with ≥98% overall agreement for all the organisms detected. The false positive/negative rate is within a reasonable range expected when testing any two methods such as is seen when PCR assay methods are compared to culture methods for all but two of the organisms. The detection sensitivity for Coronaviruses and *S. pneumoniae* by RPM v.1 is lower compared to the PCR methods and indicates the need to improve and/or optimize the primers sets used in the RPM v.1 assay for these organisms. However, the specificity remains very high for all organisms.

The ability to test for multiple pathogens is increasingly recognized as a necessity, especially in cases where differential diagnosis of etiological agents is difficult. Broad-spectrum diagnostics are particularly useful when different etiological agents that cause similar symptoms (such as respiratory illness) co-circulate in the same populations, or when co-infections of multiple organisms are of potential importance to epidemiology, symptomology, or treatment. However, it is difficult, cumbersome and costly to detect multiple pathogens and co-infections using culture methods or single test PCR methods. Currently, the reported coinfection rate among respiratory pathogens varies (4.5–23%) due to the different breadth and sensitivities of the detection methods [23]. Using RPM v.1 (and confirmed by real-time RT-PCR/PCR), this study found that 58 (13.7%) of the tested samples showed viral or viral/bacterial co-infections (Table 4). It is a challenge that will only increase in difficulty as more organisms are covered to establish the validity of any broad spectrum platform against “gold” standards as few existing archive samples have been tested for such a large number of organisms.

**Table 4:** Comparative results for specimens positive for multiple pathogens.

Organism	Culture (+)	RPM v.1 (+)
Adenovirus+Influenza A	N.C.	3
Coronavirus+Influenza A	4*	8
Coronavirus+*N. meningitides*	-	1
Influenza A+Influenza B	N.C.	3
Influenza+PIV	N.C.	1
Influenza A+*H. Influenzae*	2	N.C.
Influenza A+*M. pneumoniae*	N.C.	1
Influenza A+*N. meningitides*	N.C.	7
Influenza A+*S. pneumoniae*	1	15
Influenza B+Adenovirus	N.C.	1
Influenza B+Coronavirus	N.C.	1
Influenza B+*S. pneumoniae*	N.C.	5
Influenza B+*S. pyogenes*	N.C.	3
*N. meningitides*+*S. pneumoniae*	N.C.	2
Influenza A+*N. meningitides*+*S. pneumoniae*	N.C.	2
Influenza A+*Neisseria spp.*+*S. pneumoniae*	N.C.	1
Influenza B+*Neisseria spp.*+*S. pneumoniae*	N.C.	1
Influenza A+Influenza B+*S. pneumoniae*	N.C.	1
Influenza A+Influenza B+Coronavirus+*S. pneumoniae*	N.C.	1
Rhinovirus+PIV+*N. meningitides*+*S. pneumoniae*	N.C.	1

Note: *Coronaviruses were identified through CAP-certified PCR method, N.C.: no coinfection found, samples may have identified as positive for one of the organisms

In addition to pathogen detection, sequences obtained using the RPM v.1 provide useful additional information for tracking strain variation without additional testing. This study shows that the majority of the clinical samples tested are positive for influenza A/B virus (74%), which is not surprising for the samples collected during the flu season. Comparison of conventional sequencing for representative influenza A positive samples demonstrates the utility of this information from RPM v.1. The isolates belong to two major lineages of influenza A/H3N2 and all isolates are evolved from the A/Fujian/411/02 strain, one component of the influenza vaccine for 2004–2005 season. Antigenic drift selected by vaccination apparently leads to new strains of influenza A/H3N2 during 2004–2005. As anticipated, the RPM v.1 identifies the A/California/7/2004 lineage as the dominant circulating strain. The interesting feature is the emergence of Wellington lineage strains at the same time in significant numbers, which are of the same clade as the A/Wisconsin/67/2005 strain, a suggested vaccine component for 2006–2007 season. These results confirm that the RPM v.1 is a useful tool itself not only for influenza surveillance but also for predicting antigenic variation that will be beneficial for vaccine candidate predication. Considering all of these results, a high level of confidence in the general reliability of the resequencing approach for simultaneous pathogen detection and strain/variant identification directly from clinical samples has been established.

The results show excellent correlation with reference assays, but neither the RPM v.1 nor the tests conducted in the reference assays represent complete coverage of all respiratory pathogens. Consequently, no pathogen is detected for 14% of the specimens, but the patients still exhibit flu-like symptoms. Furthermore, the RPM v.1 is designed as a proof-of-concept microarray for the detection of more than 20 common respiratory pathogens encountered among military basic trainees with large section of the chip dedicated to adenoviruses and influenza viruses, but in this work it is used for surveying a slightly different population within Washington, D.C. metropolitan region, which have a broad age, gender, and geographic distribution and may have a different mix of common pathogens. In addition, the major causes of the common cold, rhinoviruses and enteroviruses, are under represented on the RPM v.1. Subsequently, only a few strains of the recognized 99 strains of rhinovirus and none of the enteroviruses can be detected by RPM v.1. The confirmatory assays do identify several organisms (i.e. PIV2, *H. influenzae*) in clinical specimens that the RPM v.1 is not designed to detect. Additionally, preliminary testing of a new chip design with more thorough coverage (>75 pathogens) identifies pathogens that the confirmatory assays detect but which the RPM v.1 cannot detect (unpublished data). This indicates the potential of this technology to reduce the number of samples not attributed to a tested organism to a significantly smaller number than is currently possible.

The major difficulties of the current technology are associated with the primer selection for amplification of the chosen targets. The design of the multiplex amplification strategy is a time consuming effort. The current system remains somewhat vulnerable to the rapid mutation of RNA viruses and each new resequencing array design that increases the number of pathogens would require recalibrating the multiplex primers mix. One solution that already mitigates this is the grouping of pathogens into subsets for amplifications that are recombined for chip analysis. In the future, the more variable pathogens can be partitioned into their own mix so that the performance for the conserved pathogens i.e. bacteria are not affected when primers are changed and only mixes that have targets added to them require recalibration. Simplified redesign and alternative amplification methods that provide the necessary sensitivity with more comprehensive coverage are also currently being investigated. Despite these limitations, the positive results of this study lead us to believe the resequencing microarray is an excellent candidate for the next generation pathogen detection tools and provide a modern, broad-spectrum infectious disease surveillance solution for critical decision makers, including healthcare providers, patients, public health authorities, and framers of biosecurity policy.

## Supporting Information

Table S1 (0.12 MB DOC)

Table S2 (0.03 MB DOC)

Table S3 (0.03 MB DOC)
