# Supplementary material for: Application of Broad-Spectrum, Sequence-Based Pathogen Identification in an Urban Population
Source: PLoS One. 2007 May 9;2(5):e419. doi: 10.1371/journal.pone.0000419 (PMC1855431; doi:10.1371/journal.pone.0000419)
Supplement: Table S1 — (0.12 MB DOC) [file pone.0000419.s001.doc]

Table S1. List of primers used for species specific quantitative real-time PCR

| Primer name | Sequence (5’→ 3’) | Organism  gene | Amplicon size (bp) | Accession no.  Reference |
| --- | --- | --- | --- | --- |
| AMPfor1 | GAC CAA TCC TGT CAC CTC TGA | **Influenza A**  *matrix* | 229 | AF138708  (Stone et al., 2004) |
| AMPrev1 | GTA TAT GAG KCC CAT RCA ACT |
| BMA-F1 | TCG GTG GGA AAG AAT TTG AC | **Influenza B**  *matrix* | 162 | AF100378  (Lin et al., 2006) |
| BMA-R1 | TTC CTG ATA GGG GCT CTG TG |
| Ad4F-F | ACA AGC AAG GAG ATA GCA TAG ATG | **Human Adenovirus 4**  *fiber* | 281 | X76547  (Lin et al., 2006) |
| Ad4F-R | GTA GGA GAA GGT GTA TGA GTT AGC |
| Ad5hexon-F1 | GAC GGA GCC AGC ATT AAG TTT GAT | **Human Adenovirus 5**  *hexon* | 168 | AY601635  (Vora et al., 2006) |
| Ad5hexon-R1 | GTT GGC GGG TAT AGG GTA GAG CAT |
| Ad7F-F | ACA ACT GCC TAT CCT TTC AAT G | **Human Adenovirus 7**  *fiber* | 281 | X76547  (Vora et al., 2006) |
| Ad7F-R | GAC CAA GTT ACA CGA ATA CAA TAT G |
| 229E-FP | TTC CGA CGT GCT CGA ACT TT | **Coronavirus 229E**  *membrane glycoprotein* | 222 | M33560  (Vijgen et al., 2005) |
| 229E-RP | CCA ACA CGG TTG TGA CAG TGA |
| OC43-FP | ATG TTA GGC CGA TAA TTG AGG ACT AT | **Coronavirus OC43**  *membrane glycoprotein* | 284 | M93390  (Vijgen et al., 2005) |
| OC43-RP | AAT GTA AAG ATG GCC GCG TAT T |
| PIV2s | CCA TTT ACC TAA GTG ATG GAA | **Parainfluenza 2**  *hemagglutinin* | 140 | U70948 (Templeton et al., 2004) |
| PIV2as | CGT GGC ATA ATC TTC TTT TT |
| PIV3s | GGA GCA TTG TGT CAT CTG TC | **Parainfluenza 3**  *hemagglutinin-neuraminidase* | 150 | M20402 (Templeton et al., 2004) |
| PIV3as | TAG TGT GTA ATG CAG CTC GT |
| Rhino-5’-F1 | TGC TTT ACC CAA GGC AAA AA | **Rhinovirus 89**  5’ noncoding region | 130 | NC_001617  (Lin et al., 2006) |
| Rhino-5’-R1 | AGC CTC ATC TGC CAG GTC TA |
| RS-F1 | AAC AGA TGT AAG CAG CTC CGT TAT C | **Respiratory Syncytial Virus**  *Fusion protein (F)* | 90 | AF067125  (Mentel et al., 2003) |
| RS-F2 | CGA TTT TTA TTG GAT GCT GTA CAT TT |
| RS-F3^∅^ | TGC CAT AGC ATG ACA CAA TGG CTC CT |
| OPJR | CGG GAT TCC CCG CGG AGG | ***M. pneumoniae***  *P1 adhesion protein* | 76 | X07191  (Ursi et al., 2003) |
| OPJS | CAC CCT CGG GGG CAG TCA G |
| ply894 | TGC AGA GCG TCC TTT GGT CTA T | ***S. pneumoniae***  *pneumolysin* | 81 | M17717  (Corless et al., 2001) |
| ply974 | CTC TTA CTC GTG GTT TCC AAC TTG A |
| ply941^∅^ | TGG CGC CCA TAA GCA ACA CTC GAA |
| Spy1258F | AAA GAC CGC CTT AAC CAC CT | ***S. pyogenes***  *Spy1258* | 407 | AE006565  (Liu et al., 2005) |
| Spy1258R | TGG CAA GGT AAA CTT CTA AAG CA |
| porA-F2 | CGG CAG CGT (C/T)CA ATT CGT TC | ***N. meningitidis***  *PorA (Porin)* | 309 | AF239810  (Molling et al., 2002) |
| porA-R2 | CAA GCC GCC TTC CTC ATA GC |

Note: ^∅^ Dual-labeled probe contains 6-carboxy-fluorescein (FAM) as the fluores-reporter dye at the 5’ end, and the Black Hole Quenchers at the 3’ end.

Table S1. Continued

| Primer name | Sequence (5’→ 3’) | Organism  gene | Amplicon size (bp) | Accession no.  Reference |
| --- | --- | --- | --- | --- |
| Ad5fiber-F1 | TAT TCA GCA TCA CCT CCT TTC C | **Human Adenovirus 5**  *fiber* | 2000 | AY601635  (Vora et al., 2006) |
| Ad5fiber-R1 | AAG CTA TGT GGT GGT GGG GC |
| AdCF | TGC TTG CGC THA AAA TGG GCA | **Human Adenovirus 5**  *fiber* | Ad1-630  Ad2-204  Ad5-405  Ad6-929 | (Adhikary et al., 2004) |
| Ad1R | CGA GTA TAA GAC GCC TAT TTA CA |
| Ad2R | CGC TAA GAG CGC CGC TAG TA |
| Ad5R | ATG CAA AGG AGC CCC GTA C |
| Ad6R | CTT GCA GTC TTT ATC TGA AGC A |

**References:**

Adhikary, A. K., Inada, T., Banik, U., Numaga, J., and Okabe, N. (2004): Identification of subgenus C adenoviruses by fiber-based multiplex PCR. *J Clin Microbiol* **42**, 670-3.

Corless, C. E., Guiver, M., Borrow, R., Edwards-Jones, V., Fox, A. J., and Kaczmarski, E. B. (2001): Simultaneous detection of Neisseria meningitidis, Haemophilus influenzae, and Streptococcus pneumoniae in suspected cases of meningitis and septicemia using real-time PCR. *J Clin Microbiol* **39**, 1553-8.

Lin, B., Blaney, K. M., Malanoski, A. P., Ligler, A. G., Schnur, J. M., Metzgar, D., Russell, K. L., and Stenger, D. A. (2006): Using Resequencing Microarray as a Multiple Respiratory Pathogen Detection Assay. *J Clin Microbio*.

Liu, D., Hollingshead, S., Swiatlo, E., Lawrence, M. L., and Austin, F. W. (2005): Rapid identification of Streptococcus pyogenes with PCR primers from a putative transcriptional regulator gene. *Res Microbiol* **156**, 564-7.

Mentel, R., Wegner, U., Bruns, R., and Gurtler, L. (2003): Real-time PCR to improve the diagnosis of respiratory syncytial virus infection. *J Med Microbiol* **52**, 893-6.

Molling, P., Jacobsson, S., Backman, A., and Olcen, P. (2002): Direct and rapid identification and genogrouping of meningococci and porA amplification by LightCycler PCR. *J Clin Microbiol* **40**, 4531-5.

Stone, B., Burrows, J., Schepetiuk, S., Higgins, G., Hampson, A., Shaw, R., and Kok, T. (2004): Rapid detection and simultaneous subtype differentiation of influenza A viruses by real time PCR. *J Virol Methods* **117**, 103-12.

Templeton, K. E., Scheltinga, S. A., Beersma, M. F., Kroes, A. C., and Claas, E. C. (2004): Rapid and sensitive method using multiplex real-time PCR for diagnosis of infections by influenza a and influenza B viruses, respiratory syncytial virus, and parainfluenza viruses 1, 2, 3, and 4. *J Clin Microbiol* **42**, 1564-9.

Ursi, D., Dirven, K., Loens, K., Ieven, M., and Goossens, H. (2003): Detection of Mycoplasma pneumoniae in respiratory samples by real-time PCR using an inhibition control. *J Microbiol Methods* **55**, 149-53.

Vijgen, L., Keyaerts, E., Moes, E., Maes, P., Duson, G., and Van Ranst, M. (2005): Development of one-step, real-time, quantitative reverse transcriptase PCR assays for absolute quantitation of human coronaviruses OC43 and 229E. *J Clin Microbiol* **43**, 5452-6.

Vora, G. J., Lin, B., Gratwick, K., Meador, C., Hansen, C., Tibbetts, C., Stenger, D. A., Irvine, M., Seto, D., Purkayastha, A., Freed, N. E., Gibson, M. G., Russell, K., and Metzgert, D. (2006): Co-infections of adenovirus species in previously vaccinated patients. *Emerg Infect Dis* **12**, 921-30
