# Supplementary material for: Application of Broad-Spectrum, Sequence-Based Pathogen Identification in an Urban Population
Source: PLoS One. 2007 May 9;2(5):e419. doi: 10.1371/journal.pone.0000419 (PMC1855431; doi:10.1371/journal.pone.0000419)
Supplement: Table S2 — (0.03 MB DOC) [file pone.0000419.s002.doc]

**Table S2.** Evaluation of the detection efficiency for adenovirus, coronavirus and *S. pneumoniae* in clinical samples

|  | *Adenovirus* | | *Coronavirus* | |
| --- | --- | --- | --- | --- |
|  | Ref© + | Ref© - | Ref © + | Ref © - |
| RPM v.1 + | 8 | 1 | 22 | 2 |
| RPM v.1 - | 0 | 415 | 7 | 393 |
| Sensitivity | 100 % | | 76% | |
| Specificity | 99.7 % | | 99.5% | |
| Overall agreement | 99.8 % | | 97.8% | |

Ref©: reference diagnostic methods, culture and/or PCR
