# Supplementary material for: Application of Broad-Spectrum, Sequence-Based Pathogen Identification in an Urban Population
Source: PLoS One. 2007 May 9;2(5):e419. doi: 10.1371/journal.pone.0000419 (PMC1855431; doi:10.1371/journal.pone.0000419)
Supplement: Table S3 — (0.03 MB DOC) [file pone.0000419.s003.doc]

**Table S3.** Evaluation of the detection efficiency for *S. pneumoniae* and negative in clinical samples

|  | S. pneumoniae | | S. pyogenes | | *Negative* | |
| --- | --- | --- | --- | --- | --- | --- |
|  | Ref© + | Ref© - | Ref© + | Ref© - | Ref © + | Ref © - |
| RPM v.1 + | 36 | 2 | 13 | 0 | 52 | 7 |
| RPM v.1 - | 5 | 381 | 0 | 411 | 0 | 365 |
| Sensitivity | 88% | | 100 % | | 100% | |
| Specificity | 99.5% | | 100 % | | 98% | |
| Overall agreement | 98% | | 100 % | | 98% | |
